# Targeting the Complement Pathway in Malignant Glioma Microenvironments

**DOI:** 10.3389/fcell.2021.657472

**Published:** 2021-04-01

**Authors:** Hongtao Zhu, Xingjiang Yu, Suojun Zhang, Kai Shu

**Affiliations:** ^1^Department of Neurosurgery, Tongji Hospital, Tongji Medical College, Huazhong University of Science and Technology, Wuhan, China; ^2^Department of Histology and Embryology, School of Basic Medicine, Tongji Medical College, Huazhong University of Science and Technology, Wuhan, China

**Keywords:** glioblastoma microenvironments, malignant glioma, complement pathway, immunotherapy, tumor immunity

## Abstract

Malignant glioma is a highly fatal type of brain tumor, and its reoccurrence is largely due to the ordered interactions among the components present in the complex microenvironment. Besides its role in immune surveillance and clearance under physiological conditions, the complement system is expressed in a variety of tumor types and mediates the interactions within the tumor microenvironments. Recent studies have uncovered the broad expression spectrum of complement signaling molecules in the tumor microenvironment and various tumor cells, in particular, malignant glioma cells. Involvement of the complement system in tumor growth, immunosuppression and phenotype transition have also been elucidated. In this review, we enumerate the expression and function of complement molecules in multiple tumor types reported. Moreover, we elaborate the complement pathways in glioma cells and various components of malignant glioma microenvironments. Finally, we summarize the possibility of the complement molecules as prognostic factors and therapeutic targets in the treatment of malignant glioma. Specific targeting of the complement system maybe of great significance and value in the future treatment of multi-type tumors including malignant glioma.

## Introduction

Glioblastoma treatments have been largely unsuccessful due to its rapid growth, multidimensional heterogeneity, and stubborn resistance to chemoradiotherapy (Lim et al., 2018). For decades, the main treatment for malignant glioma has been surgical resection combined with postoperative radiotherapy and chemotherapy. Unfortunately, even with the maximal treatment, the median survival time of patients with glioblastoma is still less than 15 months (McGranahan et al., 2019). Hence, new treatment strategies need to be developed.

Unlike early oncology research that focused on the proliferation and invasion of tumor cells, recent studies have shown that almost all solid tumors have a complex but highly ordered tumor microenvironment. For example, the glioma microenvironment contains glioma cells, glioma stem cells (GSCs), tumor-associated macrophages (TAMs), vascular endothelial cells, neurons, astrocytes, T cells, neutrophils, and extracellular matrix molecules. These components organically interact with each other and work together to promote tumor progression (Broekman et al., 2018). It is well known that simultaneously targeting the interaction between the components in the tumor microenvironment when inhibiting the growth of tumor cells can inhibits tumor growth and prolongs the survival of tumor-bearing mice more effectively.

As an important part of the immune system, the complement system plays an irreplaceable role in a series of pathophysiological processes, such as immune surveillance, immune clearance, and inflammatory response (Ricklin et al., 2010; Ehrnthaller et al., 2011). Recent studies have found that complement-related molecules, including C1q, C3a/C3aR, and C5a/C5aR, are expressed in the tumor microenvironment (Roumenina et al., 2019b). These molecules are widely involved in a series of tumor biological processes, such as tumor cell proliferation and invasion, resistance to radiotherapy and chemotherapy, monocyte recruitment, phenotype polarization of TAMs, and tumor neovascularization (Zhang et al., 2019). In gliomas, other reports demonstrate that the expression of complement molecules and complement signals can promote tumor progression (van der Vlis et al., 2018).

In this review, we summarized the composition and function of the complement system, the expression and related roles of complement molecules in various types of tumors, and the effects of complement molecules in the malignant glioma microenvironment. Moreover, we discussed the potential of using complement signals in the diagnosis and treatments of malignant glioma. Complement signaling could promote the growth of malignant glioma cells and the proneural to mesenchymal transition; both of these factors promote tumor progression. We believe that the complement system participates in the recruitment of peripheral blood monocytes, polarization of TAMs, neovascularization, and maintenance of GSCs. The complement system may also play a significant role in hypoxia signaling. Targeting the complement signaling pathway will become important for the comprehensive treatment of malignant gliomas in the future.

## The Complement System

**FIGURE 1:**
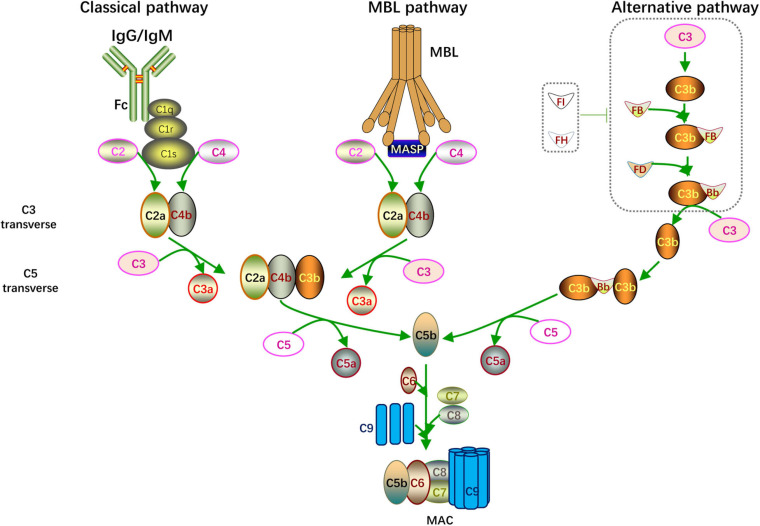
Complement activation. Overview of the complement activation. There are three different pathways including classical pathway (initiated by IgG/IgM), MBL pathway (activated when MBL binding to MASP) and alternative pathway (microorganism components enhanced C3 autohydrolyzation and accumulation of C3 transverse). The catalytic activity of C3, C5 transverse and formation of C5b-9 complex (MAC) are common feature of these pathways.

The complement system, which was first discovered and named by J. Bordet in 1890, is one of the most indispensable components of the human immune system (Morgan, 2000; Kolev et al., 2014). This system contains a series of membrane receptors and soluble proteins mainly synthesized in the liver and released into the circulatory system. Complement proteins are widely distributed in various parts of the human body, such as the blood and tissue fluids. When pathogenic microorganisms invade the body, the complement system is activated and works in conjunction with other immune components to remove foreign microorganisms via immune regulation and the direct killing of target cells (Morgan, 2000). In addition to the classical immune clearance function, several recent studies have shown that the complement system is also widely involved in a series of pathophysiological processes, such as hemolysis, intracellular homeostasis maintenance, recognition and clearance of apoptotic cells, and recruitment of inflammatory cells (Kolev et al., 2014). Three different pathways can activate the complement system, which are described in detail below (Figure 1).

### Classical Pathway

When foreign pathogenic microorganisms invade the body, the immune system produces corresponding antibodies to help eliminate the pathogen. When IgG or IgM antibodies bind to foreign microorganisms or other non-self antigens, the complement system is activated through the classical pathway. The Fc segment of the antibody recognizes and binds to C1q and changes its configuration to activate C1r and C1s. The activated C1s cleaves C4 and C2 to C4a, C4b, C2a, and C2b. Then C4b combines with C2a to form C4b2a as C3 convertase; C3 convertase cleaves C3 and combines with the cleaved C3b to form C4b2a3b as C5 convertase. C5 convertase further hydrolyzes C5 into C5a and C5b; C5a and C3a, which are formed by the previous cleavage of C3, are secreted to the extracellular matrix and function as anaphylatoxins. C5b forms a membrane attack complex (MAC) with C6, C7, C8, and multiple C9 units. The MAC perforates the host cell membranes invaded by pathogens, thereby lysing the target cells and achieving immune clearance.

### Alternative Pathway

Unlike the classical pathway that is activated in an antibody-dependent manner, the alternative pathway is activated by the microorganism components, including bacteria, endotoxins, yeast polysaccharides, and dextran. Under physiological conditions, C3 is spontaneously divided into a small amount of C3b, which combines with factor B to constitute C3bB. The alternative form of C3 convertase, C3bBb, is created when C3Bb combines with factor D and factor P; however, the whole process is suppressed by factor I and factor H. When the above pathogenic components are invading, the inhibitory effect of factor I and factor H is blocked, leading to the accumulation of C3bBb. C3bBb cleaves C3 into C3a and C3b, forming a positive feedback loop for C3 cleavage. And C3bBb combines with C3b to form C3bBb3b as C5 convertase. C5 convertase splits C5 to C5a and C5b. Eventually, the MAC is formed through the same mechanism as the classical pathway.

### Lectin Pathway

The lectin pathway is well known as the mannose-binding lectin (MBL) pathway. When pathogens containing mannose or mannosamine residues invade the body, they can be recognized and combine with MBL to activate mannose-associated serine protease (MASP). MASP is similar to C1s in the classical pathway in which it can cleave C4 and C2 to C4a, C4b, C2a, and 2b. C4b combines with C2a to form C4b2a as C3 convertase. Then C4b2a cleaves C3 and C5 in the same way as the classical pathway, and the C5b6789n MAC is formed.

### The Complement Pathway in Cancer Research

In recent years, cancer researchers have increasingly focused on the immune microenvironment of tumors. Immune microenvironment has been found in almost all types of tumors. Immune checkpoints and immunotherapy have become hot topics in cancer research. Tumors can hijack multiple components of the immune system, thereby achieving a widespread state of tumor immunosuppression. Researchers no longer focus solely on the mechanisms of malignant cell behaviors, such as tumor cell growth and invasion; rather, they have turned their attention to the interaction between tumor cells and their microenvironmental components. As mentioned earlier, the complement system is an important part of the classical immune system, and its expression in the tumor and effect on the tumor microenvironment and tumor progression has received a lot of attention. In the following sections, we will elaborate and discuss the research progress of several classical components of the complement system and the role of complement signaling pathways in tumors (Table 1).

**TABLE 1:** Complement components in tumors.

Molecules		Cancer type	Expression	Functions	References
C1q		Breast cancer, clear cell carcinoma, Glioma	high	Promotes proliferation and invasion; correlated with patient survival	Bulla et al., 2016; Mangogna et al., 2019b; Roumenina et al., 2019a
		Lung adenocarcinoma, lung squamous cell carcinoma, ovarian cancer	low	Induces apoptosis	Kaur et al., 2016; Mangogna et al., 2019a
C3/C3a/C3aR		melanoma, lung cancer, gastric cancer, colon cancer, breast cancer, pancreatic cancer	up	promotes tumor growth, metastasis, EMT, angiogenesis; regulates the microenvironment of TAMs, MDSCs, DCs, Tregs Function; prognosis biomarker	Zhang et al., 2019
C3d		lymphoma	high	marker for tumor staging and patient prognosis	Elvington et al., 2014
		Lymphoma, melanoma	low	enhance anti-tumor immunity, inhibit tumor growth in animal models	Platt et al., 2017
C4d		follicular lymphoma, astrocytoma, malignant pleural mesothelioma, esophageal squamous cell carcinoma, lung cancer, oral and oropharyngeal squamous cell carcinoma	high	promotes tumor cells growth, invasive and resistance to chemotherapy, markers for disease diagnosis and prognosis	Zhiyong et al., 2007; Makela et al., 2012; Ajona et al., 2015a,b; Klikovits et al., 2017
C5/C5a/C5aR		cervical cancer, lymphoma, lung cancer, melanoma, breast cancer, ovarian cancer, cholangiocarcinoma, gastric cancer, renal cancer, lymphoma, liver cancer, colon cancer, pancreas cancer and glioma	high	Promotes tumor cell growth, migration, invasion, EMT, angiogenesis and treatment resistance	Ajona et al., 2018; Mastellos et al., 2018; Medler et al., 2018; Wang et al., 2019
C5b-9		lymphoma, oral squamous cell carcinoma, prostate cancer	high	Promotes tumor cell growth, inhibits tumor cell apoptosis	Niculescu et al., 1997; Pilzer and Fishelson, 2005; Liu et al., 2012; Gallenkamp et al., 2018
C7		hepatocellular carcinoma	high	enhances stemness, promote tumor growth	Seol et al., 2016; de Lima et al., 2018
		prostate and esophageal cancer	low	prognosis biomarker	Oka et al., 2001; Loveridge et al., 2020
mCRPs	CD35	follicular dendritic cell sarcoma, malignant endometrioma, leukemia, bladder cancer, and nasopharyngeal carcinoma	high	prognosis biomarker	Murray et al., 2000; Srivastava and Mittal, 2009; He et al., 2012
	CD46	breast cancer, hepatocellular carcinoma, colon cancer, and multiple myeloma	high	prognosis biomarker	Simpson et al., 1997
	CD55	colon cancer, breast cancer, prostate cancer, ovarian cancer, cervical cancer, gastric cancer, hematological malignancies, and esophageal cancer	high	Promotes tumor progression	Bjorge et al., 1997; Guc et al., 2000; Loberg et al., 2006; Ikeda et al., 2008
	CD59	diffuse large B-cell lymphoma, colorectal cancer, and prostate cancer	High	prognosis biomarker	Chen et al., 2017; Zhou et al., 2018
		breast cancer	low	prognosis biomarker	Wang Y. et al., 2017
MBL-MASP		non-Hodgkin’s lymphoma, central nervous system tumors, children with acute lymphoblastic leukemia, colon cancer, glioma	high	marker for prognosis and recurrence	Kuraya et al., 2003; Ytting et al., 2005; Fisch et al., 2011
		ovarian cancer	low	prognosis biomarker	Swierzko et al., 2007
Factor B		lung cancer, astrocytoma, pancreatic ductal adenocarcinoma and squamous cell carcinoma of the skin	high	promotes tumor growth, prognosis biomarker	Zhao et al., 2006; Lee et al., 2014; Riihila et al., 2017; Kim et al., 2019
Factor D		astrocytoma cells and gastric cancer cells	NA	NA	Barnum et al., 1992; Kitano and Kitamura, 2002
Factor H		ovarian cancer, lung cancer and breast cancer	high	promotes tumor growth and immunosuppression	Honda et al., 2017; Yoon et al., 2019; Smolag et al., 2020
RGC32		colon cancer, breast cancer, ovarian cancer, gastric cancer, pancreatic cancer, esophageal cancer, prostate cancer and lymphoma	high	promotes the proliferation, invasion, epithelial-mesenchymal transition (EMT) of tumor cells and prompts a poor prognosis	Fosbrink et al., 2005; Zhu et al., 2012; Wang X.Y. et al., 2017
		glioblastoma, astrocytoma, multiple myeloma, and adrenocortical tumors	low	Inhibits tumor growth	Vlaicu et al., 2019
		non-small cell lung cancer	high or low	undetermined	Kim D.S. et al., 2011; Sun et al., 2013; Xu et al., 2014; Zhang et al., 2020

#### C1q

As the starting molecule of the classical activation pathway, Clq can bind to the Fc segment of antibodies and initiate the complement system activation cascade. A variety of human cells, including monocytes/macrophages, epithelial cells, mesenchymal cells, dendritic cells, trophoblast cells, endothelial cells, and microglia, express C1q. C1q is also involved in various pathophysiological processes, such as trophoblast infiltration, placental development, eclampsia, and some autoimmune diseases. In the development of tumors, C1q can function as a tumor promoter or tumor suppressor (Mangogna et al., 2019a). C1q is highly expressed in Melanoma, breast cancer, clear-cell renal-cell carcinoma, and glioma (Bulla et al., 2016; Mangogna et al., 2019b; Roumenina et al., 2019a). It can promote malignant behaviors, such as tumor cell proliferation and invasion, and its expression level is negatively correlated with patient survival (Mangogna et al., 2019b; Roumenina et al., 2019a). However, the expression of C1q in lung adenocarcinoma and lung squamous cell carcinoma is lower than that in the corresponding normal tissue (Mangogna et al., 2019a). In ovarian cancer cell lines, C1q can induce apoptosis through the tumor necrosis factor (TNF) pathway (Kaur et al., 2016).

#### C3/C3a/C3aR

C3 is a key component of all three complement activation pathways. When the complement system is activated, C3 can be cleaved into C3a by C3 convertase. C3a is a small molecule composed of 77 amino acids, and work as anaphylatoxin. C3a can be secreted to the outside of the cell and bind to the C3aR receptor (C3aR) on the membrane of the target cell. C3aR is a G protein-coupled receptor that regulates various downstream signaling pathways and affects cellular activities (Ajona et al., 2019). At present, the upregulation of C3a/C3aR has been observed in melanoma, lung cancer, gastric cancer, colon cancer, breast cancer, and pancreatic cancer. Its upregulation can promote tumor growth, metastasis, epithelial-to-mesenchymal transition (EMT), and angiogenesis and regulate the microenvironment of TAMs, Myeloid-derived suppressor cells (MDSCs), dendritic cells (DCs), as well as the function of regulatory T cells (Tregs). C3a/C3aR can also be used to predict the prognosis of various tumor patients (Zhang et al., 2019).

#### C3d

When C3 convertase cleaves C3 into C3a and C3b, C3b binds to the immune complex and is further cleaved to form IC3b and C3f. Then iC3b is processed into C3c and C3dg, and C3dg decomposes into C3d and C3g (Dempsey et al., 1996). Some scholars found that C3d enhances antitumor immunity independently of B cells, natural killer cells (NK cells), or antibodies, but it does so by increasing tumor infiltrating CD8^+^ lymphocytes, by depleting Tregs, and by suppressing expression of programmed cell death protein 1 (PD-1) by T cells, which hint the potential of C3d to be used as a marker for tumor staging and patient prognosis in lymphoma (Elvington et al., 2014). Also, other scholars have confirmed that C3d can enhance antitumor immunity and significantly inhibit tumor growth in animal models of lymphoma and melanoma (Platt et al., 2017).

#### C4d

C4d is the product of C4 cleavage when the complement system is activated via the classical pathway. C4b contains a highly unstable thioester bond, which can be covalently combined with the surrounding hydroxyl or amino acid-containing molecules to form an ester bond or an amide bond. The covalently bonded C4b is hydrolyzed into C4c and C4d; C4d, which retains the thioester site, can interact with type IV collagen in the basement membranes of capillaries and form stable covalent combinations with endothelial cells (Sapir-Pichhadze et al., 2015). C4d, which has been widely reported in the immune rejection of organ transplantation and related immune diseases, can be used as a specific marker for humoral immune rejection (Nickeleit and Mihatsch, 2003; Miranda Correa et al., 2013). C4d is highly expressed in follicular lymphoma, astrocytoma, malignant pleural mesothelioma, esophageal squamous cell carcinoma, lung cancer, and oral and oropharyngeal squamous cell carcinoma. Its expression is linked with the rapid growth and invasiveness of tumor cells, their resistance to chemotherapy, and poor patient prognosis (Zhiyong et al., 2007; Makela et al., 2012; Klikovits et al., 2017). In lung cancer patients, the concentration of C4d in bronchoalveolar lavage fluid and plasma may be used as a diagnostic and prognostic marker (Ajona et al., 2015b). In patients with oral and oropharyngeal squamous cell carcinoma, salivary C4d levels can also be used to indicate patient prognosis (Ajona et al., 2015a).

#### C5/C5a/C5aR

Like C3, C5 is an indispensable component of the complement system. Under the action of C5 convertase, C5 can be hydrolyzed into a 74-amino-acid peptide (C5a) that also has an anaphylatoxin effect. It can be secreted to the outside of the cell to bind to the corresponding G protein-coupled receptor (C5aR1) of the target cell (Cain and Monk, 2002). C5a also binds to another receptor named C5aR2 (C5L2). Although C5aR2 is a seven-transmembrane protein, it is not a G protein-coupled receptor. At present, the downstream effects of C5a binding are still unclear. Researchers found that C5aR2 competitively inhibits the binding between C5a and C5aR1 based on current reports (Cain and Monk, 2002; Ward, 2009). C5a and C5aR1 are also highly expressed in a variety of tumors, such as cervical cancer, lymphoma, lung cancer, melanoma, breast cancer, ovarian cancer, cholangiocarcinoma, gastric cancer, renal cancer, lymphoma, liver cancer, colon cancer, pancreatic cancer, and glioma. It also promotes malignant behaviors including tumor cell growth, migration, invasion, EMT, angiogenesis, and treatment resistance through mechanisms like C-X-C Motif Chemokine Ligand 16 (CXCL16) and T cell response regulation (Ajona et al., 2018; Mastellos et al., 2018; Medler et al., 2018; Wang et al., 2019).

#### C5b-9

C5b-9, also known as MAC, is the final product of the complement system activation which directly perforates the target cell membrane to lyse the target cell. The traditional view is that C5b-9 can lyse target cell, inhibiting the occurrence and development of tumors (Tegla et al., 2011). However, multiple membrane-bound complement regulatory proteins (mCRPs) are expressed in tumor cells. Under the action of these proteins, the MAC cannot form a complete permeable pore across the cell membrane and becomes a partially dissolved form, sublytic C5b-9(sC5b-9), which is embedded in the membrane. sC5b-9 can activate multiple signaling pathways, including PI3K-Akt, ERK, JAK1-STAT3, and NF-κB, and regulates tumor cell growth in a G protein-dependent manner (Vlaicu et al., 2019). sC5b-9 is highly expressed in lymphomas and oral squamous cell carcinoma cells (Niculescu et al., 1997; Pilzer and Fishelson, 2005; Gallenkamp et al., 2018). In prostate cancer, sC5b-9 also inhibits TNFα-induced apoptosis (Liu et al., 2012).

#### C7

C7 combines with C6, C8, C9, and C5b to form MAC, which is indispensable for complement-mediated cell lysis. The main function of C7 is to rivet the complex on the target cell membrane. Approximately 50% of serum C7 is synthesized by the liver, and it can also be synthesized by monocytes, macrophages, lymphocytes, fibroblasts, and certain cells in the central nervous system (Gonzalez et al., 2003). The low expression of C7 in prostate and esophageal cancer corresponds to poor prognosis (Oka et al., 2001; Loveridge et al., 2020). Interestingly, in hepatocellular carcinoma, C7 enhances the stemness of hepatocellular carcinoma cells and promotes tumor growth (Seol et al., 2016; de Lima et al., 2018).

#### mCRPs

mCRPs, which include CD35, CD46, CD55, and CD59, prevent the excessive activation of complement under physiological conditions and ensure that the complement system plays a role in clearing target cells when the activation is required (Zipfel and Skerka, 2009). Recent studies have shown that the abnormal expression of mCRPs in various tumors makes them promising diagnostic markers and therapeutic targets for various tumors (Yan et al., 2008; Geller and Yan, 2019). The research findings regarding the role of each mCRP in tumors are described below.

#### CD35

CD35, or its synonyms CR1 (complement receptor 1), is a transmembrane protein widely expressed on the surface of hematopoietic cells. CD35 participates in the cleavage of C3b into iC3b *in vivo* and combines with C4b to promote its degradation and accelerate the inactivation of C3 and C5 convertases (Morgan, 2000). These steps prevent the excessive activation of the complement system. CD35 is highly expressed in follicular dendritic cell sarcoma, malignant endometrioma, leukemia, bladder cancer, and nasopharyngeal carcinoma; its high expression in nasopharyngeal carcinoma patients indicates a poor prognosis (Murray et al., 2000; Srivastava and Mittal, 2009; He et al., 2012).

#### CD46

CD46, also called membrane cofactor protein, is a transmembrane glycoprotein expressed on the surface of all nucleated cells. It is indispensable for the protection of normal tissues during complement activation. CD46 can assist complement factor I in cleaving C3b and C4b *in vivo* to avoid overactivation of the complement system (Morgan, 2000). CD46 is also involved in sperm–egg binding during fertilization and T cell activation (Kemper et al., 2003). Abnormally high expression of CD46 is observed in breast cancer, hepatocellular carcinoma, colon cancer, and multiple myeloma and is negatively correlated with patient prognosis (Simpson et al., 1997).

#### CD55

CD55, also known as decay acceleration factor, can combines with C4b and C3b to inhibit the catalysis of C2 and factor B to form C3a and Bb, thus blocking the activation of the complement system (Ward et al., 1994). Studies have shown that CD55 is abnormally expressed in colon cancer, breast cancer, prostate cancer, ovarian cancer, cervical cancer, gastric cancer, hematological malignancies, and esophageal cancer and participates in the malignant progression of these tumors (Ward et al., 1994; Bjorge et al., 1997; Simpson et al., 1997; Guc et al., 2000; Loberg et al., 2006; Ikeda et al., 2008).

#### CD59

CD59 inhibits the polymerization of multiple C9 and competitively bind C5b with C8, ultimately inhibiting the formation of MAC and lysis of target cells *in vivo*. It is vital to the body’s self-protection under physiological conditions. CD59 expression is abnormally high in diffuse large B-cell lymphoma, colorectal cancer, and prostate cancer, and its high expression suggests a poor patient prognosis (Chen et al., 2017; Zhou et al., 2018). However, Wang Y. et al. (2017) found that the high expression of CD59 in breast cancer correlates with better patient prognosis.

#### MBL–MASP

Mannose-binding lectin and mannose-associated serine protease constitute the most important starting components of the lectin pathway for complement activation. MBL and MASP2 are highly expressed in non-Hodgkin’s lymphoma, central nervous system tumors, and pediatric cases of acute lymphoblastic leukemia (Fisch et al., 2011). The blood levels of MASP2 can be used as prognostic markers for colon cancer. High MASP2 levels in the blood suggest a poor prognosis and high risk of recurrence (Ytting et al., 2005). Notably, MASP1 and MASP3 are highly expressed in the C6 and T98G glioma cell lines (Kuraya et al., 2003; Pagliara et al., 2018), but their expression is low in some ovarian cancer patients and the MASP1 and MASP3 expression levels are positively correlated with patient prognosis (Swierzko et al., 2007).

#### Factors B and D

Both factors B and D are important for the alternative pathway of complement activation. Factor B, which is mainly synthesized by the liver and macrophages, plays a significant role in tissue damage and inflammation (Campbell et al., 1984). Factor B is highly expressed in lung cancer, astrocytoma, pancreatic ductal adenocarcinoma, and squamous cell carcinomas of the skin. It also promotes the growth of squamous cell carcinoma of the skin and may be used as a predictor for the prognosis of pancreatic ductal adenocarcinoma (Zhao et al., 2006; Lee et al., 2014; Riihila et al., 2017; Kim et al., 2019). Factor D, which is also known as a lipid-lowering hormone, is a lipid-derived protein that participates in the alternative pathway of complement activation. Factor D regulates energy balance and fat metabolism via acylation-stimulating protein, which is a complement protein closely related to energy balance and fat metabolism (Song et al., 2016). Factor D is expressed in astrocytoma cells and gastric cancer cells, but its involvement in tumorigenesis and development still needs further investigation (Barnum et al., 1992; Kitano and Kitamura, 2002).

#### Factor H

The key enzymes for C3b synthesis can be hydrolyzed by Factor H, a soluble inhibitor of the complement system. Under physiological conditions, it is combined with its own components and exerts inhibitory effect during complement activation. By exerting inhibitory effects, it prevents excessive activation that damages the normal tissues. When the gene encoding factor H is mutated, it cannot recognize “self” components, thus causing the abnormal activation of complement. Tumors may also hijack the function of factor H to achieve immune evasion (Parente et al., 2017). Studies have shown that factor H can form a complex with fibrin γ to promote disease progression in the blood of ovarian cancer patients (Honda et al., 2017). Factor H is also highly expressed in lung cancer and breast cancer and promotes tumor growth via immunosuppression (Yoon et al., 2019; Smolag et al., 2020).

#### RGC32

Response gene to complement 32 (RGC32) was identified after sublytic complement activation, and it is widely expressed in the liver, kidney, skeletal muscle, and other tissues (Badea et al., 1998). RGC32 is involved in cell differentiation, cell cycle regulation, and immune regulation under physiological conditions (Badea et al., 1998; Vlaicu et al., 2008). In tumors, the expression and function of RGC32 are disease-dependent (Vlaicu et al., 2019). RGC32 is highly expressed in colon cancer, breast cancer, ovarian cancer, gastric cancer, pancreatic cancer, esophageal cancer, prostate cancer, and lymphoma. Moreover, it promotes the proliferation, invasion, and EMT of tumor cells and is associated with poor prognosis (Fosbrink et al., 2005; Zhu et al., 2012; Wang X.Y. et al., 2017). However, RGC32 is downregulated in glioblastoma, astrocytoma, multiple myeloma, and adrenocortical tumors and acts as a tumor suppressor instead (Vlaicu et al., 2019). Interestingly, RGC32 can be both upregulated and downregulated in non-small-cell lung cancer, and its specific expression and function still need more in-depth revelation in the context of this disease (Kim D.S. et al., 2011; Sun et al., 2013; Xu et al., 2014; Zhang et al., 2020).

## The Complement Pathways in Glioma Microenvironments

**FIGURE 2:**
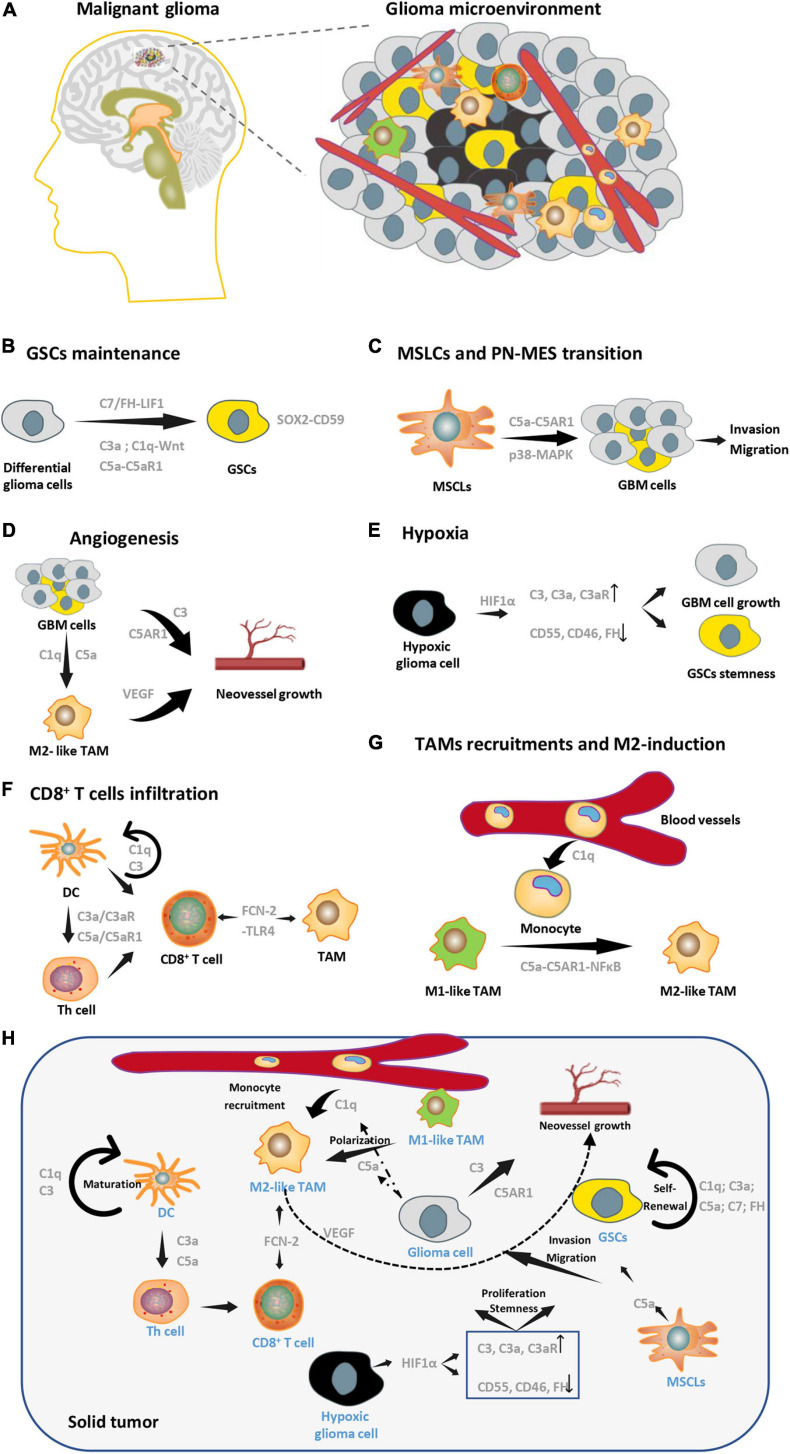
Complement pathways in glioma microenvironment. **(A)** A brief schematic diagram of glioma microenvironments. **(B)** Complement molecules (C3a, C1q, C5a, and C7) can promote GSCs stemness maintenance. **(C)** C5a secreted by MSLCs enhances invasion and migration of GBM cells. **(D)** Complement pathways can enhance angiogenesis by targeting both tumor cells and M2-TAMs in GBM. **(E)** Expression patterns of complement molecules change in hypoxia, which facilitates GSCs stemness and GBM growth. **(F)** C3 and C1q are essential for DC’s maturation, maturated DC can regulate the function of Th cell by C3a-C3aR and C5a-C5aR1 axis, which affect the infiltration of CD8^+^ T cells. FCN2 can direct target TAM and CD8^+^ T cell through its receptor, TLR4. **(G)** C1q and C5a are essential in recruitment of peripheral monocytes and M2 polarization of macrophages, respectively. **(H)** A conclusion of multicellular interactions in glioma microenvironments via complement signaling.

Gliomas are the most prevalent and highly refractory tumors in the adult central nervous system. Glioblastoma multiform (GBM) representing approximately 57% of all gliomas, which have a high degree of malignancy (Tan et al., 2020). Even after receiving the maximum surgical resection within the safety range supplemented with standard postoperative radiotherapy and chemotherapy, the median survival time of patients with GBM is still less than 15 months (McGranahan et al., 2019). The poor prognosis of GBM patients is attributed to highly aggressive tumor cells and their resistance to radiochemotherapy. Solid glioblastoma tumors contain stromal cells, such as monocytes/macrophages, microglia, T cells, neurons, astrocytes, oligodendrocytes, vascular endothelial cells, and mast cells (Broekman et al., 2018). These cells and the extracellular matrix components together form a microenvironment that interacts organically with the tumor (Figure 2A). Current studies on the glioblastoma microenvironment mainly focus on the hypoxic microenvironment, perivascular microenvironment, and immune microenvironment that, composed of tumor-associated macrophages/microglias. As mentioned above, the complement system plays a significant role in the promotion or inhibition of tumor growth, and this is also true for gliomas. In the following sections, we will elaborate on research findings focusing on the complement system and its role in glioma cells and the glioma microenvironment (Table 2).

**TABLE 2:** Complement components in Glioma.

Molecules	Expression	Functions	Evidence	References
C1q (three chain, C1qA C1qB C1qC)	up-regulated (tumor tissue)	unfavorable prognostic marker	Database (Oncomine UALCAN CGGA) IHC	Mangogna et al., 2019b
C1q	up-regulated (Patient Serum and tumor tissue)	N/A	ELISA IHC IF	Bouwens et al., 2015
Factor B	down-regulated (Patient Serum)	N/A	ELISA	Bouwens et al., 2015
MBL	up-regulated (Patient Serum)	N/A	ELISA	Bouwens et al., 2015
C3	up-regulated (tumor tissue)	N/A	IHC IF	Bouwens et al., 2015
	up-regulated (Cell line)	Promotes tumor cell proliferation and invasion	EdU Transwell	Li et al., 2014
RGC32	Down-regulated (Cell line)	Inhibits tumor cell mitosis and proliferation	WB qRT-PCR Colony-forming assay	Saigusa et al., 2007
C5aR1	up-regulated (Patient specimen and cell line)	unfavorable prognosis Promotes tumor cell invasion and migration	Database (TCGA) Transwell 3D invasion assay	Lim et al., 2020
C4d	up-regulated (Patient specimen)	unfavorable prognosis	IHC(Tissue- microarray)	Makela et al., 2012
Factor H	up-regulated (Patient CSF)	N/A	proteomic analysis	Gahoi et al., 2018

### Research on the Complement Pathway in Glioma Cells

#### C1q

As the initial recognition component, C1q plays a significant role in the classical activation pathway of complement. Several studies show that C1q is expressed in various tumor microenvironments and widely involved in tumor progression. Bouwens et al. (2015) found the concentration of C1q increased in the blood of glioma patients. Next, Mangogna et al. searched the Oncomine, UALCAN, and CGGA databases and found that the *C1QA, C1QB*, and *C1QC* genes, which encode the three chains of C1q, were all highly expressed in gliomas. Furthermore, the high levels of expression for these three genes were associated with poor prognosis. These conclusions were verified by immunohistochemistry and immunofluorescence (Mangogna et al., 2019b).

#### C3a/C3aR

C3a is a small protein secreted originally from C3 cleavage, which can exert its biological functions when combined with its receptor on the surface of target cells through autocrine or paracrine signaling in various tumors (Morgan, 2000). At present, the mechanism of the C3a/C3aR signaling pathway and the expression of its components have not been elucidated in the context of gliomas. However, several studies have shown that human astrocytoma cell lines can express and secrete C3a, and the expression is regulated by IL-1β, TNFα, and IFN-γ (Barnum et al., 1993; Barnum and Jones, 1995). Nevertheless, its detailed function still needs further study.

#### C5a/C5aR1

C5a is the most active anaphylatoxin involved in multiple pathophysiological processes. C5a usually binds with its receptor (C5aR1) to mediate downstream signaling pathways and biological effects. As described, the C5a/C5aR1 axis is widely expressed in tumors, such as cervical cancer, lymphoma, lung cancer, and glioma, and participates in disease occurrence and development. Gasque et al. (1995) have identified C5aR1 on the surface of astrocytoma cells as early as 1995. C5L2, another C5a receptor, was also identified on the surface of astrocytes by Gavrilyuk et al. (2005). However, the detailed function of C5a and the conditions required for it to bind to each receptor have not been elucidated. Lim et al. confirmed that C5a is highly expressed in the Mesenchymal stem-like cells (MSLCs) of glioma microenvironment and promotes a series of malignant behaviors, such as invasion and migration of glioma cells and GSCs through its receptor C5aR1 *in vitro* and *in vivo*. MSLC-secreted C5a increases ZEB1 expression via activation of p38 MAPK in GBM cells, thereby enhancing the invasion of GBM cells into parenchymal brain tissue (Lim et al., 2020). This significantly promotes the growth of tumors and shortens the survival time of tumor-bearing mice. These findings suggest that the C5a/C5aR1 axis plays a significant role in the development of gliomas and the interaction of glioma cells with their microenvironmental components (Lim et al., 2020).

#### RGC32

RGC32 is a complement response gene induced by the MAC. It interacts with Akt and cyclin B1-CDC2 complex and is involved in cell cycle activation (Tegla et al., 2011). When the RGC32 protein was identified, the researchers revealed that its overexpression promotes DNA synthesis in the oligodendrocyte-C6 glioma hybrid cell (Badea et al., 1998). Other groups showed that compared with normal brain tissue, the protein and mRNA levels of RGC32 are low in several glioma cell lines, including T98G, U251, U373, and U87 (Fernandez-Aroca et al., 2019). They also found that mRNA expression of RGC32 is inversely correlated with the tumor grade of the cell line origin; this phenomenon is especially notable in p53 mutant tumors (Saigusa et al., 2007). RGC32 overexpression can significantly inhibit the growth of glioma cells. Also, RGC32 aggregates in the centrosomes of glioma cells during mitosis and can be phosphorylated *in vitro* by forming a protein complex with PLK1. This suggests that p53-induced RGC32 expression in gliomas inhibits mitosis and thereby inhibits tumor cell growth (Imoto et al., 2007). However, some researchers reached the opposite conclusion. Li et al. (2014) found that RGC32 is highly expressed in the U251 glioma cell line. Its overexpression can promotes proliferation and invasion of U251 cells but has no impact on apoptosis, however, its silencing enhances cell apoptosis and attenuates proliferation and invasion of U251 cells (Li et al., 2014). Since the above two studies were limited to cell lines, more in-depth investigation in patient specimens, primary cell lines, and animal experiments is required to determine the expression of RGC32 in gliomas and its mechanism in tumorigenesis and disease development.

### Complement Pathways in the Glioma Microenvironment

#### Glioma Stem Cells (GSCs)

Glioma stem cells, a small subpopulation of tumor cells endowed with sustained clonogenic potential and high tumorigenic capability, are among the root causes of refractory tumors and recurrence (Lathia et al., 2015). GSCs was firstly identified by Singh, SK, and Houman in 2003 (Hemmati et al., 2003; Singh et al., 2003, 2004) and interacts with differential glioma cells, TAMs and hypoxic conditions of glioma microenvironments (Zhou et al., 2015; Shi et al., 2017; Man et al., 2018; Wang et al., 2018). Multiple studies have shown that complement signaling plays a significant role in maintaining the stemness of cancer stem cells. For example, C7 and factor H are highly expressed in hepatocellular carcinoma stem cells and stimulate LSF-1 expression, which is localized in the nucleus and binds to the promoters of the Nanog, Oct4, Sox2, and c-Myc genes. This leads to the upregulation of the stemness factors, which finally increases cancer stemness (Seol et al., 2016). The stem cell-specific transcription factor SOX2 upregulates the expression of the complement regulatory protein CD59 in epithelial tumor stem cells to help them avoid immune surveillance of the complement system (Chen et al., 2017). C5a promotes hematopoietic stem cell activation through C5aR1 signaling (Bujko et al., 2017). Although there is no direct evidence that the complement signaling pathway affects the stemness of GSCs, it has been confirmed that the differentiation and migration of neural stem cells under physiological conditions are regulated by C3a (Shinjyo et al., 2009). Furthermore, complement C1q activates canonical Wnt signaling and promotes aging-associated decline in tissue regeneration, which is important for the maintenance of the stemness of various tumor stem cells, including gliomas (Naito et al., 2012; Sadahiro et al., 2018; Figure 2B).

#### Mesenchymal Stem-Like Cells (MSLCs)

Mesenchymal stem cells (MSCs) refer to pluripotent stem cells derived from bone marrow that do not have a hematopoietic function. Under physiological conditions, MSCs exhibit tropism to tissue injury and participate in the renovation of damaged tissue (Bianco et al., 2008). The expression of molecular markers similar to MSCs and MSLCs occurs in several tumor stromata, including that of GBM (Kim S.M. et al., 2011; Ho et al., 2012). After being recruited to the glioma microenvironment, MSCs are remodeled into MSLCs, which are important tumor-related stromal cells. Lim et al. (2020) found that MSLCs in the glioma microenvironment express and secrete high levels of C5a, which increases the expression of ZEB1 through C5aR1 and the activation of the p38-MAPK pathway in a paracrine manner. This signaling pathway promotes the invasion and migration of GBM cells, which relates to the prognosis of GBM patients (Lim et al., 2020; Figure 2C).

#### Endothelial Cells

Gliomas undergo extremely rapid growth, which is accompanied by vigorous neovascularization and the formation of incompletely functioning microvessels. In addition to providing nutrients for tumor growth, these vascular endothelial cells also facilitate organic interactions with other components of the tumor microenvironment in order to promote tumor growth. In ovarian cancer, inhibition of C3 or C5aR reduces the expression of vascular endothelial growth factor (VEGF) and neovascularization (Nunez-Cruz et al., 2012). C1q and C5a are involved in the recruitment of monocytes and the M2 polarization of TAMs which indirectly promote neovascularization through VEGF secretion (Benoit et al., 2012; Piao et al., 2018). Contrarily, C3a and C5a do not promote neovascularization in lung cancer and cervical cancer, which may be due to the different microenvironments and activation of various signaling pathways in different types of tumors (Markiewski et al., 2008; Corrales et al., 2012). Whether complement systems participate in glioma neovascularization needs further study. In addition to angiogenesis, complement signals also mediate the interaction between vascular endothelial cells and other microenvironmental components. Several studies have shown that endothelial cells promote the maintenance of GSC stemness through transforming growth factor-beta (TGFβ), NO, and other signaling pathways (Sharma and Shiras, 2016). The C5a/C5aR1 axis activates the TGFβ/Smad pathway in epithelial injury of pulmonary fibrosis, and the activation of NO is blocked after silencing the expression of C3 and C5 (Montalto et al., 2003; Gu et al., 2014). Therefore, complement signals are likely to play a significant role in the interaction between vascular endothelial cells and surrounding GSCs (Figure 2D).

#### Hypoxia

A highly hypoxic environment and necrosis are two of the most prominent features of glioma, especially glioblastomas, which are characterized by “pseudopalisading” necrosis (Colwell et al., 2017). Due to the rapid growth of the tumor, the blood vessels in the tumor are mostly incompletely functioning deformed blood vessels. Therefore, the inside of the tumor is always in a state of hypoxia. However, unlike normal cells that slowly grow under long-term hypoxic conditions and eventually die, many tumors, including gliomas, adapt to hypoxia by changing their main metabolic mode to glycolysis. Tumors can further use a series of signaling pathways that are activated during hypoxia, which are represented by hypoxia-inducible factor 1-alpha (HIF1α), to promote tumor growth (Broekman et al., 2018). Studies have shown that various complement signaling molecules interact with the hypoxic microenvironment. Under hypoxia, the expressions of CD55, CD46, and factor H are downregulated by HIF1α. Meanwhile, C3, C3a, and C3aR are upregulated during hypoxia, which leads to an increase in C3a/C3aR signaling (Greijer et al., 2005; Pedersen et al., 2007). The expression of signal transducer and activator of transcription 3 (STAT3), which is upstream of HIF1α, can also regulates complement signaling expression during hypoxia (Mantovani, 2010). STAT3 plays a significant role in a series of biological processes, such as the maintenance of GSC stemness under hypoxic conditions in gliomas (Qiang et al., 2012; Figure 2E).

#### Tumor-Associated Macrophages (TAMs)

Tumor-associated macrophages that account for about 30–40% of the total cells in solid gliomas are the most abundant immune cells in the glioma microenvironment (Broekman et al., 2018). TAMs in gliomas are composed of *in situ* microglial cells and monocytes/macrophages recruited from peripheral blood under the action of multiple chemokines. TAMs can be divided into two phenotypes: the M1 like phenotype promotes immunity and inhibits tumor growth, whereas the M2 like phenotype is immunosuppressive and promotes tumor progression. In the glioma microenvironment, TAMs are mostly dominated by the M2 like phenotype, and the conversion of M1 like TAMs to the M2 like phenotype occurs constantly. M2 like TAMs secrete a variety of anti-inflammatory cytokines that inhibit the immune response and interact with tumor cells and other microenvironmental components to promote tumor progression in multiple dimensions, which is also an important reason for glioma refractory (Hambardzumyan et al., 2016). Studies clear that C1q can participate in the recruitment of peripheral blood monocytes, suppress macrophage inflammation and inflammasome activation (Benoit et al., 2012), and C5a can activate the NF-κB pathway through the C5aR1 receptor and promote the M2 like polarization of TAMs, thereby inhibiting the tumor immune response and promoting tumor progression (Piao et al., 2018). Conversely, TAMs in tumors can also regulate the expression of the complement components of the surrounding cells by secreting a series of cytokines. Although currently the role of complement signaling in glioma TAMs is largely unclear, we believe that there is an organic interaction between TAMs and the complement system to jointly promote tumor progression in gliomas (Figure 2G).

#### CD8^+^ T Cell

CD8^+^ T cells are included in the group of cells known as effector T cells. They are cytotoxic and can lyse target cells, including tumor cells. CD8^+^ T cells need to undergo maturation and activation to exert their cytotoxicity. First, CD3-T cell receptor on the surface of T cells recognizes the endogenous antigens (tumor antigens), which are bound by MHCI and presented by antigen-presenting cells (APCs) (Seder and Ahmed, 2003). With the help of helper T (Th) cells, a double-activated signal is formed, and the naïve T cell proliferates and differentiates into cytotoxic CD8^+^ T cells. These CD8^+^ T cells are responsible for lysing tumor cells under the action of IL-2 and other cytokines (Boyman and Sprent, 2012). Studies have shown that the activation of T cells is widely affected by complement signals. First, the maturation of DCs, which plays a major role in antigen presentation, depends on complement molecules C1q and C3 (Peng et al., 2006; Baruah et al., 2009). Second, the binding of C3a and C5a to C3aR and C5aR1, respectively, promotes DC cell and Th cell function through monocytes, which finally facilitate CD8^+^ T cell activation (Lalli et al., 2008). Furthermore, Treg cells also express C3aR and C5aR1 that, can inhibit their function after activation and indirectly promote T cell activation (van der Touw et al., 2013). However, studies have also shown that the complement system inhibits T cell activation and induces immune tolerance (Pio et al., 2019). C1q can inhibit the proliferation of Th cells in a DC-dependent manner (Benoit et al., 2012). When iC3b binds to its receptor CR3 on the surface of APCs, immunosuppressive cytokines, such as TGF-β2 and IL-10, are secreted, thus inhibiting the antigen presentation process (Sohn et al., 2003). CD46 also promotes the binding of endogenous C5a to the receptor C5aR2 to negatively regulate Th cell activity (Kemper et al., 2003). The effect of complement signaling on the regulation of CD8^+^ T cell infiltration also differs between tumors. The inhibition of C5AR1 expression in squamous cell carcinoma can promote intratumoral CD8^+^ T cell infiltration (Medler et al., 2018). Fatty Acid Binding Protein 5 (FABP5) is highly correlated with complement signaling in uveal melanoma, and it promotes CD8^+^ T cell infiltration (Xu et al., 2020). C3 knockout mice-bearing lung cancer exhibited increased numbers of CD8^+^ T cells and decreased tumor growth (Kwak et al., 2018). Ding et al. (2017) believe the serum complement component Ficolin-2 (FCN-2) in various cell lines, including colon cancer and lung cancer. FCN-2 combines with Toll-like receptor 4 (TLR4) on the surface of macrophages and DC cells to inhibit tumor growth by promoting M1 like phenotypic polarization and infiltration of CD8^+^ T cells (Ding et al., 2017). In malignant gliomas, Zhang et al. clarified that the suppression of the complement system inhibited CD8^+^ T cell infiltration in IDH-mutated gliomas. They also found that the tumor metabolite D2-HG significantly inhibits the classical and alternative pathways of complement activation while inhibiting T cell activation (Zhang et al., 2018). These data suggest a correlation between complement signal and CD8^+^ T cell infiltration in malignant gliomas; however, its specific mechanism still needs further study (Figure 2F).

#### Complement Signals Function in Glioma Immune Evasion

For a long time, the body’s central nervous system was considered to be “immune escapable” compared with peripheral organs. As the most commonly diagnosed central nervous system tumor in adult patients, gliomas are mainly composed of *in situ* microglial cells. However, more recent studies have subverted this point (Lim et al., 2018). Besides *in situ* microglia, there are multiple immune components in the brain under physiological conditions that exhibit persistent immune responses (Broekman et al., 2018). Louveau et al. (2015) demonstrate that the dural venous sinuses can function as lymphatic egress, and deep cervical lymph nodes can function as “transit stations” for a series of immune cells, including B cells and T cells, involved in the immune function of the brain. Due to the abnormally rapid growth of glioma cells and the existence of abundant microvessels with immature functions and structures, the blood–brain barrier is no longer intact. Therefore, peripheral blood mononuclear cells can be recruited into tumors via chemotaxis (Zhou et al., 2015). It has been reported that the components of immune cells in glioblastomas include microglia, monocytes/macrophages derived from peripheral blood, T cells, DCs, and neutrophils (Broekman et al., 2018). Microglia/macrophages are the predominant immune cell type; they account for approximately 30% of all the cellular components of solid tumors (Broekman et al., 2018). Importantly, the above immune cells are no longer able to effectively lyse tumor cells; instead, they promote tumor progression and immunosuppression in glioblastoma under the induction of various factors from the microenvironment. The complement signaling pathway plays a role in the immune surveillance of the central nervous system under physiological conditions and during glioblastoma-induced immunosuppression. As mentioned above, several complement components, including C1q, C3a/C3aR, C5a/C5aR1, CD46, and CD55, affect the functional status of DCs, Th cells, and T cells. C1q and C5a/C5aR1 signals promote the recruitment of monocyte/macrophages from the peripheral blood and M2 phenotypic polarization (Markiewski et al., 2008; Ajona et al., 2019; Roumenina et al., 2019a). We believe that complement signals that are widely involved in the physiological immune state of the central nervous system and malignant gliomas will be assignable targets in elucidating immune signaling in malignant gliomas.

#### Complement Signaling Mediates Multicellular Interactions in Glioma Microenvironments

As described above, a number of complement molecules like C1q, C5, and C3 are highly expressed in glioma cells, and are secreted into the microenvironment to participate in polarization of TAM, recruitment of peripheral monocyte and neoangiogenesis. In addition, C1q, C3a, C5a, C7, and FH are related to self-renewal of cancer stem cells (CSC). C5a secreted by MSLCs promotes the invasion and migration of glioma cells via p38-MAPK-ZEB1 axis. C1q, C3, and C5a are essential for DC maturation and Th cell function which combine with FCN2 to regulate CD8^+^ T cell activity. Complement components expression of glioma cells in hypoxic microenvironment also altered due to transcriptional regulation of HIF1α. Hypoxic tumor cells have upregulated expression of C3-C3a-C3aR axis and downregulated CD55, CD46, and FH, which play important roles in tumor cell proliferation and CSCs stemness maintenance. To sum up, molecules of the complement pathway are mostly upregulated in glioma compared to normal brain tissue, and activated complement signaling participates in various interactions between glioma cells and its microenvironments, promoting the glioma growth (Figure 2H).

## Therapeutic Opportunities

### Diagnosis

As shown above, the complement signaling pathway plays a significant role in the interaction between the various components of the malignant glioma microenvironment and promotes tumor progression. Complement signaling molecules can be used as targets for the early diagnosis of gliomas. Studies have shown that the C1q and MBL concentrations in the blood of glioma patients are higher and the concentration of factor B is lower compared with those of healthy volunteers (Bouwens et al., 2015). These suggest that early screening of the above factors in the patients’ blood contributes to the diagnosis and prognostic judgment of glioma patients. And, the brain tumors represented by malignant gliomas are accompanied by specific changes in the properties of cerebrospinal fluid. Recently, Gahoi et al. (2018) found higher levels of factor H in the cerebrospinal fluid of glioma patients compared with those of healthy volunteers through proteomics analyses. The concentration of factor H is directly proportional to the tumor grade (Gahoi et al., 2018), thus indicating that changes in the levels of complement molecules in the cerebrospinal fluid may be used for disease diagnosis.

### Treatment Strategy

Since complement signals participate in a variety of biological processes related to tumor progression, they are potentially excellent drug targets for comprehensive tumor therapy. Inhibiting complement signals can synergistically block multiple biological processes. Wang et al. (2020) found that inhibiting C3b/C4b while performing anti-vascular therapy can inhibit neovascularization, cell proliferation, MDSC infiltration and promote tumor cell apoptosis in the tumor microenvironment. It is also worth mentioning that the inhibitors of complement signals act in a relatively systematic manner to the in-depth investigation of complement signaling in non-tumor immune-related diseases, such as organ transplantation. There are at least 30 inhibitors or neutralizing antibodies targeting 14 complement signaling molecules that are currently undergoing clinical trials (Riihila et al., 2019), as represented by eculizumab, an inhibitor of C5, recently has been reported to effectively re-establishes regulation of the innate immune complement system to substantially reduce the pathophysiological manifestations of human CHAPLE disease (Ozen et al., 2021) and IPH5401, a C5aR neutralizing antibody was combined with Durvalumab to treat Patients with advanced solid tumors (STELLAR-001) (Clinical trail, NCT number: 03665129). All of the above descripted suggest that in numerous tumors, including malignant gliomas, targeting complement signaling pathways may become a new, effective, and easy treatment strategy in the future.

## Conclusion

Although a lot of studies have revealed the role of complement signaling pathways in the development of a variety of tumors, complement components, and complement-related proteins are a large family of proteins, and their detailed mechanisms in tumor pathogenesis and applications in treatment strategies still need to be further elucidated. As mentioned above, complement signals widely impact tumor cells and several microenvironmental components in gliomas. Further revealing its detailed mechanism of action will certainly help the future comprehensive treatment of gliomas and improve patient prognosis.

## Author Contributions

KS and SZ designed the study. HZ and XY wrote and finished the manuscript. All authors contributed to the article and approved the submitted version.

## Conflict of Interest

The authors declare that the research was conducted in the absence of any commercial or financial relationships that could be construed as a potential conflict of interest.
